# Discordance Between Systemic Lupus Erythematosus Disease Activity Index Domain Weights and Their Association With Organ Damage Accrual

**DOI:** 10.1002/acr.80077

**Published:** 2026-05-12

**Authors:** Kevin Zhang, Eric F. Morand, Sarah E. Boyd, François Petitjean, Alberta Hoi, Rachel Koelmeyer, Iolanda Miceli, Julie A. Monk, Hieu T. Nim

**Affiliations:** ^1^ Centre for Inflammatory Diseases Monash University Clayton Victoria Australia; ^2^ Data Science & AI, Faculty of Information Technology Monash University Clayton Victoria Australia; ^3^ The Novo Nordisk Foundation Centre for Stem Cell Medicine (reNEW), Murdoch Children's Research Institute Melbourne Victoria Australia

## Abstract

**Objective:**

Studies of damage accrual in patients with systemic lupus erythematosus (SLE) show associations with disease activity measured by the SLE Disease Activity Index 2000 (SLEDAI‐2K), but these associations are imperfect. SLEDAI scores are powerfully influenced by weightings (1–8) assigned to each domain. We investigated whether the weightings of SLEDAI‐2K domain scores aligned with the risk of organ damage accrual.

**Methods:**

In patients with SLE attending a single center from 2007 to 2016, disease activity (SLEDAI‐2K) and organ damage (Systemic Lupus International Collaborating Clinics/American College of Rheumatology damage index; SDI) were recorded prospectively at each visit and annually, respectively. Visits were dichotomized according to whether the visit occurred during a period of damage transition (increase in SDI in the same year). Associations were assessed using Fisher exact test (univariable analysis) and generalized linear mixed‐effect models (multivariable analysis).

**Results:**

We analyzed 5,538 visits of 266 patients (86.5% female, 51.5% White). SLEDAI‐2K domains positively associated with damage transition all had weights of ≤4 (odds ratio [95% confidence interval]: pericarditis, 5.39 [1.59–18.3] *P* < 0.01; pleurisy, 2.12 [1.14–3.94] *P* = 0.02; arthritis, 1.40 [1.02–1.94] *P* = 0.04; and leukopenia, 1.52 [1.04–2.24] *P* = 0.03). Higher‐weighted SLEDAI‐2K domains were negatively associated with damage transition, not associated, or were too infrequent to assess.

**Conclusion:**

Associations of SLEDAI‐2K domains with damage accrual were variable and were discordant with domain weightings. Alternative weighting strategies for disease activity instruments could improve their prognostic utility.

## INTRODUCTION

Systemic lupus erythematosus (SLE) is the archetypal multisystem autoimmune disease. Accrual of irreversible organ damage, for example as measured by the Systemic Lupus International Collaborating Clinics/American College of Rheumatology (SLICC/ACR) Damage Index for SLE (SDI),^1^ is an important outcome for patients with SLE and is associated with increased morbidity and mortality.^2^ A key treatment goal in SLE, therefore, is to prevent organ damage accrual, a goal which is usually sought by controlling disease activity.^3^ As such, understanding the association between disease activity measurements and a patient's risk of organ damage accrual is of value.

**SIGNIFICANCE & INNOVATIONS**

- In systemic lupus erythematosus (SLE), SLE Disease Activity Index 2000 (SLEDAI‐2K) total scores are modestly predictive of organ damage accrual, but domain weightings that determine total scores have not been validated based on association with damage accrual.
- This study aimed to evaluate SLEDAI‐2K domain weightings for their associations with organ damage accrual in SLE.
- We found that the weightings of SLEDAI‐2K organ domains were discordant with organ damage accrual risk, with lower‐weighted SLEDAI‐2K domains positively associated with damage and higher‐weighted domains negatively or not associated with damage transition.
- This finding suggests that damage prediction in SLE could be improved by refinement of disease activity measures via data‐driven weightings based on association with outcomes.

The multisystem nature of SLE complicates disease activity measurement systems. The SLE Disease Activity Index (SLEDAI), a well‐established scoring system created in 1992^4^ and revised in 2002 (SLEDAI‐2K),^5^ sums scores in 24 weighted domains and provides an overall assessment of disease activity at a point in time. Although some studies have demonstrated positive associations between SLEDAI‐2K score and organ damage,^6^ others show weak or nonsignificant associations.^2^, ^7^, ^8^ The different weightings (1–8) of SLEDAI domains, assigned using expert consensus methodology with ranking of each domain's relative importance,^4^ means that total SLEDAI scores are inescapably affected by domain weightings. Thus, domain weightings have an impact on the ability of SLEDAI to predict a patient's risk of damage accrual. However, whether the domain weights in SLEDAI are optimal for SLEDAI to be used to predict damage accrual has not been carefully studied. To determine if weightings of domains in SLEDAI‐2K are “correct” vis a vis the prediction of organ damage, we evaluated associations between individual SLEDAI‐2K domain scores and organ damage accrual over time, using univariable and multivariable analyses in a longitudinal cohort of patients with SLE.

## PATIENTS AND METHODS

### Participants and data collection

Longitudinal clinical data were collected from patients with SLE attending a single center (Monash Lupus Clinic, Melbourne, Victoria, Australia) between May 2007 and December 2016. Patients were aged ≥18 years and fulfilled the 1982 ACR revised criteria,^9^ as described.^10^ Patients self‐reported race and ethnicity based on a fixed set of categories. At each visit, disease activity was scored using the SLEDAI‐2K,^5^ and clinical laboratory tests results and details of medication use, including daily dose of glucocorticoid (prednisolone or equivalent), were recorded. Organ damage (SDI) was assessed annually, and only patients with at least two consecutive annual measures of SDI were included in this study. All enrolled patients gave written informed consent, and this study was approved by the Human Research Ethics Committee, Monash Health (reference 15526L) in accordance with the Declaration of Helsinki. Neither patients nor the public were involved in the design or implementation of this study.

### Damage accrual transition status

In this and many other cohorts, disease activity is measured at every visit but damage once per year. To associate visit data with damage accrual, each 1‐year period between SDI measures was classified as a “damage transition” period if there was an increase in the SDI score between the previous and subsequent annual visits and a “nondamage transition” period if there was no increase. Accordingly, visits that occurred in these periods were assigned as “damage transition” or “nondamage transition” visits, as follows. By denoting each annual visit as *T*_*i*_ where *i* = 1…*n*, the damage transition state $\lambda \left(t\right)$ for each routine visit *t* was defined as follows:

$$\lambda \left(t\right)=\left(\mathrm{SDI}\left(T_{i+1}\right)-\mathrm{SDI}\left(T_{i}\right),1\right),\;\text{where}\;T_{i}\leq t<T_{i+1}$$

Each visit with $\lambda \left(t\right)=0$ was labeled as “nondamage transition” and each visit with $\lambda \left(t\right)=1$ was labeled as “damage transition.”

### Statistical analysis

The association of disease activity at each visit in each SLEDAI domain (rescored unweighted, as present or absent) and prednisolone use (present or absent) with that visit being a damage transition or nondamage transition visit was analyzed using a two‐tailed Fisher exact test for the univariable analysis and a generalized linear mixed‐effects model for the multivariable analysis. We developed a generalized linear mixed‐effects model with a logistic link function and a linear predictor function as follows:

$$f\left(X\right)=\beta^{T}X+e+z_{i},$$

where X was the vector of explanatory variables (all SLEDAI domains and prednisolone), β was a vector of the regression estimates of each element X, T was the matrix transpose,e was the fixed random effect, and $z_{i}$ was the unknown random effects with respect to patient *i*. This mixed‐effects model allowed us to forego the assumption of independence by adding a random intercept $z_{i}$ for each patient, which assumes that patients respond uniformly to a change in explanatory variable. Maximum likelihood estimation was used to estimate the unknown parameters β with the R software environment (3.4.0) using the package “lme4”^11^ and the Bound Optimization BY Quadratic Approximation optimization algorithm^12^ until convergence.

## RESULTS

### Baseline demographics and disease characteristics

In total, 5,538 patient visits from 266 patients were analyzed (median number of visits per patient 18 [interquartile range (IQR) 2–28]). Patients were predominantly female, of Asian or White race and ethnicity, and had a median age of 38 years, a median disease duration of 5 years, and a median SLEDAI‐2K of 4 at enrollment (Table 1). During follow‐up (median 4.9 [IQR 2.8–7.9] years), 41.7% of patients (111 of 266) had organ damage accrual.

**Table 1:** Patient demographics and disease characteristics at enrollment*

Demographics/disease characteristics	Patients (N = 266)
Age, median (IQR), y	38 (28–49)
Sex, n (%)	
Male	36 (13.5)
Female	230 (86.5)
Ethnicity and race, n (%)	
White	136 (51.5)
Asian	79 (29.7)
Other	51 (18.8)
Disease duration, median (IQR), y	5.0 (1.8–10.6)
SLEDAI‐2K, median (range)	4 (0–26)
SDI, median (range)	0 (0–4)
Medications, n (%)	
Glucocorticoids	132 (49.6)
Hydroxychloroquine	156 (58.6)
Methotrexate	20 (7.5)
Azathioprine	47 (17.6)
Serology, n (%)	
ANA	258 (97.0)
Anti‐dsDNA	175 (65.8)
Low complement	103 (38.7)
Antiphospholipid antibodies	17 (6.4)

* ANA, antinuclear antibodies; ds, double‐stranded; IQR, interquartile range; SDI, Systemic Lupus International Collaborating Clinics/American College of Rheumatology Damage Index; SLEDAI‐2K, Systemic Lupus Erythematosus Disease Activity Index 2000.

### SLEDAI‐2K domains associated with organ damage accrual

In the univariable analysis, damage transition was statistically significantly associated with disease activity in several SLEDAI‐2K domains (rash, alopecia, pleurisy, pericarditis, fever, urinary casts, hematuria, proteinuria, pyuria, low complement, increased DNA binding, arthritis, and leukopenia) with odds ratios (ORs) ranging from 1.16 (fever) to 6.81 (pericarditis). (Supplementary Table S2). All other SLEDAI‐2K domains (thrombocytopenia, mucosal ulcers, myositis, visual disturbance, vasculitis, seizure, psychosis, organic brain syndrome, lupus headache, cranial nerve disorder, and stroke) were not associated with damage transition. Prednisolone use was significantly associated with damage transition (OR 2.19 [95% confidence interval (CI) 1.88–2.56]; *P* < 0.001).

After multivariable analysis, fewer statistically significant associations between SLEDAI‐2K domain activity and damage transition were retained (Figure 1, Table 2; Supplementary Table S2 [raw output]). Pericarditis (OR 5.39 [95% CI 1.59–18.3]; *P* < 0.01), pleurisy (OR 2.12 [95% CI 1.14–3.94]; *P* = 0.02), leukopenia (OR 1.52 [95% CI 1.04–2.24]; *P* = 0.03), arthritis (OR 1.40 [95% CI 1.02–1.94]; *P* = 0.04), and prednisolone use (OR 1.65 [95% CI 1.28–2.10]; *P* < 0.001) were significantly associated with damage transition. In contrast, vasculitis (OR 0.471 [95% CI 0.239–0.930]; *P* = 0.03) and cranial nerve disorder (OR 0.204 [95% CI 0.0425–0.986]; *P* < 0.05) were negatively associated with damage transition. None of the highest‐weighted SLEDAI domains (visual disturbance, vasculitis, seizure, psychosis, organic brain syndrome, lupus headache, cranial nerve disorder, or stroke) had a positive association with damage transition in this model.

**Figure 1:**
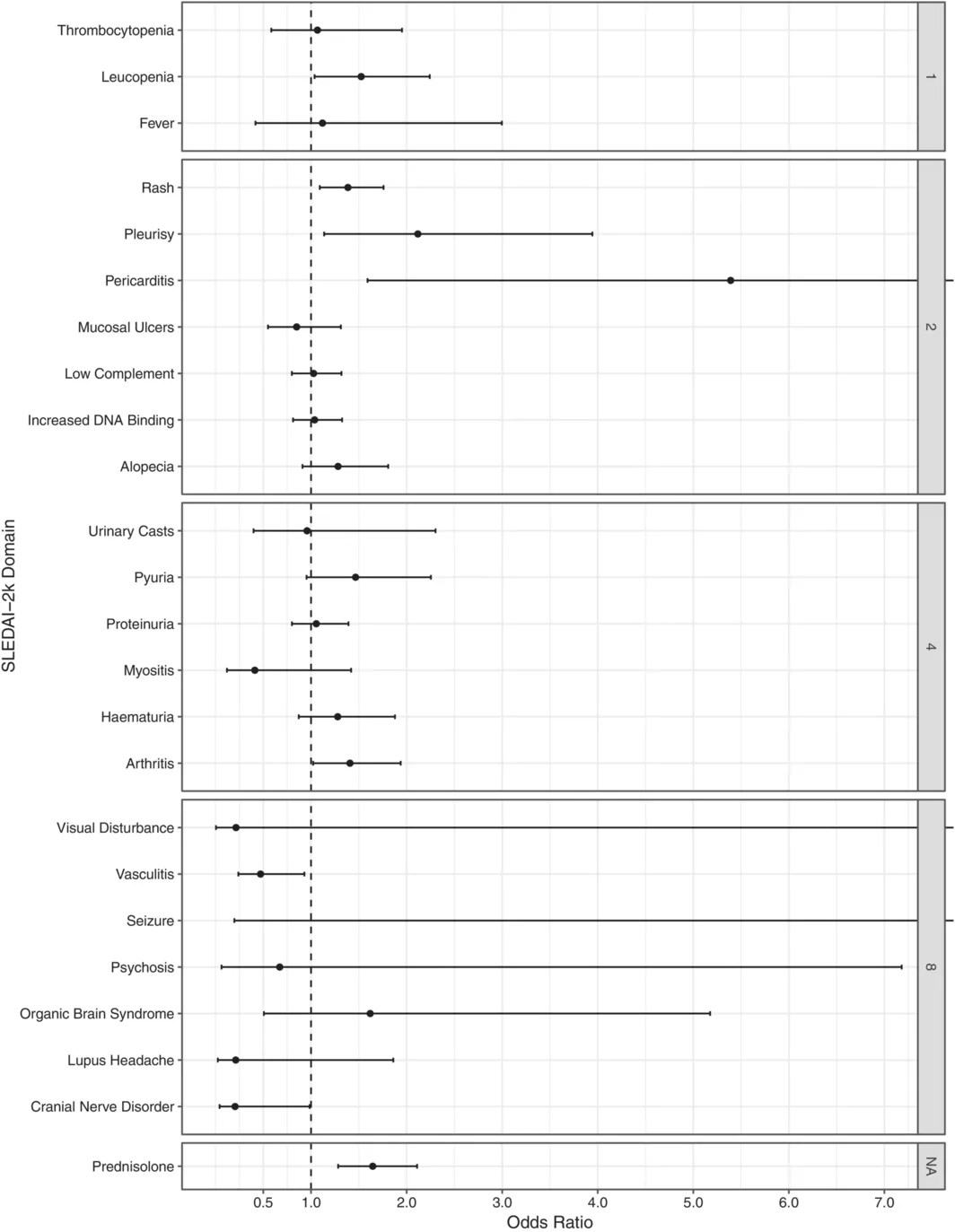
Multivariable linear mixed‐effects regression analysis of the association of activity in SLEDAI‐2K domains with damage transition. The odds ratio and 95% confidence intervals are presented for each SLEDAI‐2K domain (excluding stroke because only one case was recorded) and prednisolone use. Disease activity domains are grouped according to domain weight. Visits were classified as damage transition visits if they occurred during a damage transition period (increase in Systemic Lupus International Collaborating Clinics/American College of Rheumatology Damage Index score between the previous and subsequent annual visits). NA, not applicable; SLEDAI‐2K, Systemic Lupus Erythematosus Disease Activity Index 2000.

**Table 2:** Multivariable analysis of the association of Systemic Lupus Erythematosus Disease Activity Index 2000 domains with damage transition*

Domain (domain weight)	Transition, n	Nontransition, n	OR (95% CI)	*P*
Leukopenia (1)	71	192	1.52 (1.04–2.24)	0.03
Pleurisy (2)	30	73	2.12 (1.14–3.94)	0.02
Pericarditis (2)	13	8	5.39 (1.59–18.3)	<0.01
Arthritis (4)	120	273	1.40 (1.02–1.94)	0.04
Vasculitis (8)	16	55	0.471 (0.239–0.930)	0.03
Cranial nerve disorder (8)	3	18	0.204 (0.0425–0.986)	<0.05

* *P* < 0.05 is significant. CI, confidence interval; OR, odds ratio.

## DISCUSSION

Accrual of irreversible organ damage in SLE is associated with increased morbidity and mortality.^2^ The ability to predict the risk of organ damage accrual in individual patients is highly desirable to assign preventive treatment. In the research setting, the ability to predict organ damage risk could be used to study interventions designed to lower that risk. In formal cohort studies the association of damage risk with disease activity measured with current tools is variable or modest. The most commonly used disease activity measure, SLEDAI‐2K, is generally recorded and analyzed as a total score in which the varied weights assigned to different domains have a marked effect on the total score. We hypothesized that these weights may have unintended consequences on the ability of SLEDAI‐2K measurements to predict damage risk. Thus, we evaluated whether the SLEDAI‐2K organ domain weightings align with organ damage risk. By analyzing per visit SLEDAI‐2K domain activity and annual SDI scores in a longitudinal SLE cohort, we found that the weightings of SLEDAI‐2K domains are not congruent with organ damage risk.

Overall, we found variable associations between SLEDAI‐2K domain activity and damage accrual. Significant positive associations were detected in several low‐weighted domains, specifically pericarditis, pleurisy, leukopenia, and arthritis, suggesting that disease activity in these domains could provide prognostic information and be further examined as potential triggers for intervention. Unexpectedly, no association between higher‐weighted SLEDAI‐2K domains and organ damage risk was found. Vasculitis is assigned the maximum weight (ie, 8) for a SLEDAI‐2K domain, yet our results revealed a negative association between vasculitis and damage transition; at the very least, no positive association could be demonstrated. Similarly, arthritis and urinary casts have the same SLEDAI‐2K domain weight (ie, 4), yet arthritis was positively associated with damage transition whereas urinary casts were not. Similar dissonance between SLEDAI‐2K domain weights and damage transition was seen in multiple other domains, such that the rank order of domain weightings did not align with damage risk. Although the gravity of disease manifestations such as proteinuria or vasculitis, which require treatment escalation, is not in doubt, our findings failed to support the current weighting of SLEDAI‐2K domains in terms of their association with clinical outcomes.

We demonstrated a positive association between glucocorticoid use and damage accrual, concordant with previous studies.^2^ Surprisingly, however, we failed to demonstrate a corresponding association between vasculitis and central nervous system (CNS) disease with damage accrual, even though these disease manifestations typically require high‐dose glucocorticoid treatment. One possible explanation for this finding is that manifestations such as arthritis and serositis often necessitate prolonged glucocorticoid treatment. In contrast, vasculitis and CNS disease, although serious, tend to be episodic in nature. Thus, the effect on damage accrual of cumulative exposure to glucocorticoids over time may differ from the effects of shorter‐term high‐dose treatment. We did not examine the impact of treatments aside from glucocorticoids in association with damage transition; this decision was driven by our previous analysis in which we did not see significant associations of immunosuppressant use with damage accrual.^13^

A key treatment goal in SLE is the prevention of organ damage accrual, which is achieved, in part, by reducing disease activity to defined thresholds, such as the lupus low disease activity state.^10^, ^14^ Importantly, the SLEDAI‐2K was developed as a descriptive rather than a predictive tool, and, accordingly, the weights applied were not determined a priori on the basis of their association with future outcomes. Our data suggest that the SLEDAI‐2K domain weights are not proportional to the association of activity in a given domain with damage accrual. In addition, for any given SLEDAI‐2K score, numerous permutations of SLEDAI‐2K domain activities are possible. Therefore, patients with similar SLEDAI‐2K scores may have different odds of damage accrual because each SLEDAI‐2K domain has a different association with organ damage accrual. The discordance between SLEDAI‐2K domain weightings and organ damage may explain previous findings of the modest or nonsignificant associations between SLEDAI‐2K score and damage accrual.^2^, ^7^, ^8^ Ultimately, these findings suggest the need for a different approach to disease activity measurement for the purposes of predicting the risk of damage accrual.

This study had some limitations. First, the analysis of individual visits assumed independence between visits. Second, the lack of events in some SLEDAI‐2K domains that are less frequent, such as neurologic domains, hindered assessment of the association of these domains with organ damage accrual risk. In addition, we did not capture organ‐specific damage, meaning that we were not able to ascertain how disease activity in a domain predicted the development of organ damage in the corresponding organ system. The frequency of which SDI domains were most affected was not reported in this study, as we considered the number of domain‐specific damage events to be too small to allow this to be meaningfully analyzed. Importantly, limitations in the SDI to detect low levels of damage have been acknowledged in recent years, with efforts to improve the SDI currently under way.^15^ This raises the possibility that the discordance between SLEDAI‐2K domain weightings and SDI may be partially attributable to limitations in the SDI rather than the weightings of SLEDAI‐2K domains. Lastly, a larger sample size might have shown significance for SLEDAI‐2K domains that did not survive multivariable adjustment in our model.

In conclusion, our results show that the associations between SLEDAI‐2K domains and the risk of damage accrual are variable and discordant with domain weights. Future studies could assess the association of SLEDAI‐2K domain weightings with other important clinical outcomes and evaluate the need for reweighting of SLEDAI‐2K domains to improve damage risk prediction in SLE.

## AUTHOR CONTRIBUTIONS

All authors contributed to at least one of the following manuscript preparation roles: conceptualization AND/OR methodology, software, investigation, formal analysis, data curation, visualization, and validation AND drafting or reviewing/editing the final draft. As corresponding author, Dr Morand confirms that all authors have provided the final approval of the version to be published and takes responsibility for the affirmations regarding article submission (eg, not under consideration by another journal), the integrity of the data presented, and the statements regarding compliance with institutional review board/Declaration of Helsinki requirements.

## Supporting information


**Disclosure form**.

**Supplementary Table 1:** Univariable analysis of the association of SLEDAI‐2K domains with damage transition
**Supplementary Table 2:** Raw regression output from the generalized linear models mixed‐effects model
