# Supplementary material for: Discordance Between Systemic Lupus Erythematosus Disease Activity Index Domain Weights and Their Association With Organ Damage Accrual
Source: Arthritis Care Res (Hoboken). 2026 May 12;78(11):1663–8. doi: 10.1002/acr.80077 (PMC13710774; doi:10.1002/acr.80077)
Supplement: Supplementary file 2 [file ACR-78-1663-s002.docx]

**Supplementary Table 1.** Univariable analysis of the association of SLEDAI-2K domains with damage transition

| **Domain (domain weight)** | **Transition (n)** | **Non-Transition (n)** | **OR (95% CI)** | ***P*** |
| --- | --- | --- | --- | --- |
| Thrombocytopenia (1) | 30 | 94 | 1.33 (0.85–2.04) | 0.17 |
| Leukopenia (1) | 71 | 192 | 1.57 (1.16–2.09) | **<0.01** |
| Fever (1) | 12 | 19 | 2.64 (1.16–5.74) | **0.01** |
| Rash (2) | 251 | 646 | 1.70 (1.44–2.02) | **<0.01** |
| Pleurisy (2) | 30 | 73 | 1.72 (1.08–2.68) | **0.02** |
| Pericarditis (2) | 13 | 8 | 6.81 (2.61–18.9) | **<0.01** |
| Mucosal ulcers (2) | 46 | 142 | 1.36 (0.95–1.92) | 0.09 |
| Low complement (2) | 684 | 2,450 | 1.43 (1.24–1.64) | **<0.01** |
| Increased DNA binding (2) | 621 | 2,411 | 1.16 (1.01–1.33) | **0.03** |
| Alopecia (2) | 92 | 200 | 1.99 (1.52–2.59) | **<0.01** |
| Urinary casts (4) | 18 | 32 | 2.35 (1.23–4.34) | **0.01** |
| Pyuria (4) | 119 | 154 | 3.48 (2.68–4.49) | **<0.01** |
| Proteinuria (4) | 305 | 882 | 1.61 (1.37–1.87) | **<0.01** |
| Myositis (4) | 9 | 17 | 2.21 (0.86–5.25) | 0.08 |
| Hematuria (4) | 154 | 300 | 2.32 (1.87–2.86) | **<0.01** |
| Arthritis (4) | 120 | 273 | 1.93 (1.52–2.42) | **<0.01** |
| Visual disturbance (8) | 1 | 7 | 0.592 (0.01–4.61) | 1 |
| Vasculitis (8) | 16 | 55 | 1.21 (0.64–2.15) | 0.55 |
| Seizure (8) | 2 | 1 | 8.30 (0.43–49) | 0.1 |
| Psychosis (8) | 1 | 10 | 0.414 (0.01–2.91) | 0.7 |
| Organic brain syndrome (8) | 7 | 15 | 1.94 (0.67–5.07) | 0.17 |
| Lupus headache (8) | 1 | 10 | 0.414 (0.01–2.92) | 0.7 |
| Cranial nerve disorder (8) | 3 | 18 | 0.69 (0.13–2.37) | 0.78 |
| Stroke (8) | 1 | 0 | NA (0.11–Inf) | NA |

CI = confidence interval; NA = not applicable; OR = odds ratio; SLEDAI-2K = systemic lupus erythematosus Disease Activity Index 2000

**Supplementary Table 2.** Raw regression output from the generalized linear models mixed-effects model

|  | **Estimate** | **SE** | **z value** | **Pr(>\|z\|)** |
| --- | --- | --- | --- | --- |
| (Intercept) | -4.11 | 0.34 | -12.1 | <0.01 |
| Seizure | 2.94 | 2.33 | 1.26 | 0.21 |
| Psychosis | -0.4 | 1.21 | -0.33 | 0.74 |
| Organic brain syndrome | 0.48 | 0.59 | 0.81 | 0.42 |
| Visual disturbance | -1.54 | 1.88 | -0.82 | 0.41 |
| Cranial nerve disorder | -1.59 | 0.8 | -1.98 | 0.05 |
| Lupus headache | -1.55 | 1.11 | -1.4 | 0.16 |
| Vasculitis | -0.75 | 0.35 | -2.17 | 0.03 |
| Arthritis | 0.34 | 0.16 | 2.09 | 0.04 |
| Myositis | -0.89 | 0.63 | -1.41 | 0.16 |
| Rash | 0.33 | 0.12 | 2.68 | 0.01 |
| Alopecia | 0.25 | 0.17 | 1.42 | 0.16 |
| Mucosal ulcers | -0.16 | 0.22 | -0.74 | 0.46 |
| Pleurisy | 0.75 | 0.32 | 2.36 | 0.02 |
| Pericarditis | 1.68 | 0.62 | 2.71 | 0.01 |
| Fever | 0.11 | 0.5 | 0.22 | 0.82 |
| Urinary casts | -0.04 | 0.45 | -0.1 | 0.92 |
| Hematuria | 0.25 | 0.2 | 1.25 | 0.21 |
| Proteinuria | 0.05 | 0.14 | 0.38 | 0.71 |
| Pyuria | 0.38 | 0.22 | 1.74 | 0.08 |
| Low complement | 0.03 | 0.13 | 0.2 | 0.84 |
| Increased DNA binding | 0.04 | 0.13 | 0.29 | 0.77 |
| Thrombocytopenia | 0.06 | 0.31 | 0.21 | 0.83 |
| Leukopenia | 0.42 | 0.2 | 2.14 | 0.03 |
| Prednisolone | 0.5 | 0.13 | 3.92 | <0.01 |

SE = standard error
